# Characteristics of silence among Chinese people in intercultural communication: a proceduralized grounded theory analysis

**DOI:** 10.3389/fpsyg.2026.1840234

**Published:** 2026-05-21

**Authors:** Hongya Fan, Weiqian Xiang

**Affiliations:** School of Foreign Languages, Shanxi University, Taiyuan, Shanxi, China

**Keywords:** Chinese people, intercultural communication, multicultural environments, proceduralized grounded theory, silence

## Abstract

**Introduction:**

While silence is a pivotal communicative resource in Chinese culture, existing research often remains fragmented and static, failing to capture its dynamic evolutionary mechanisms in intercultural encounters. This study aims to move beyond essentialist stereotypes by investigating the pragmatic characteristics and underlying psychological processes of Chinese silence. It addresses how silence is strategically negotiated and how it evolves from a situational choice into a habitual response in intercultural settings.

**Methods:**

Adopting a proceduralized grounded theory approach, the study utilized constant comparative analysis to inductively develop a theoretical framework. Data were gathered from multi-source authentic intercultural interactions, including semi-structured interviews, naturalistic observations, and textual artifacts. This methodology allowed for a participant-driven exploration of the lived experiences and strategic agency of interlocutors.

**Results:**

The analysis identifies a dynamic ecosystem of intercultural silence consisting of four interrelated stages: Culture-Driven Predispositions provide the normative foundation for silence, which is then recalibrated through Context-Mediated Negotiations; this adaptation enables Strategic Enactment, and the perceived effectiveness of such enactments fosters Ego-Protective Inertia, reinforcing silence as a habitual response.

**Discussion:**

These findings reframe Chinese silence as dynamic cultural semiotics rather than a mere communicative void. The study contributes to intercultural communication theory by integrating situational adaptability with identity-linked habit formation. Practically, the results provide diagnostic tools for distinguishing strategic silence from passive absence, offering valuable insights for enhancing intercultural sensitivity and fostering mutual understanding in increasingly multicultural environments.

## 1 Introduction

Silence, as a form of non-verbal communication and a semiotically dense resource, carries diverse meanings depending on the cultural context (Nakane, 2007; Sun, 2016; Gutiérrez and Paniagua, 2024; Hayati and Sinha, 2024). Chinese communicative silence constitutes a culturally intentional praxis rather than a void in discourse. As Hall (1976) characterized, Chinese high-context culture relies on indirect cues and shared understandings, where silence serves as a pragmatic cue for conveying deference, thoughtfulness, or relational harmony. This cultural coding, however, becomes a common source of friction and conflict in Sino-Western communication, with accumulating comparative studies confirming the pervasiveness of systemic misinterpretation. While Chinese culture frames silence as a manifestation of respect, conflict avoidance, and implicit approval, Western culture (particularly among native English speakers) tends to construe silence as an indicator of indifference, hostility, disagreement, or limited linguistic proficiency (Dodu-Savca, 2022; Gutiérrez and Paniagua, 2024; Gu, 2004). Such divergent cultural underpinnings of silence often lead to misperceptions of Chinese communicative silence, undermining interaction effectiveness and interpersonal relationship building. These misunderstandings may arise from silence duration, contextual appropriateness, and semantic meanings in interactions (Lemak, 2012; Dodu-Savca, 2022). For instance, Zhu and Bresnahan (2018) found that Western teachers and students often hold negative perceptions of Chinese students' silence, misattributing it to inadequate language proficiency or low classroom participation. Similarly, Verouden et al. (2018) documented that in Sino-Dutch academic collaborations, Dutch partners consistently interpreted their Chinese counterparts' silence as withdrawal or disengagement, eroding mutual trust and impeding project progress. This suggests a paradox: When Chinese interactants employ contextually appropriate silence, their intercultural counterparts frequently misattribute such behavior to passivity or incompetence. This underscores how Chinese silence in intercultural settings, filtered through the lens of disparate cultural norms, can yield unintended negative evaluations and outcomes.

Extant research on Chinese silence has primarily advanced along two trajectories: cross-cultural comparative studies and scenario-specific empirical analyses. Notwithstanding these insights, current scholarship remains largely fragmented and static, offering limited understanding of the dynamic, evolutionary mechanisms of silence. Existing frameworks often fail to encapsulate the complexity of silence as a contextually adaptive and strategically enacted practice, neglecting the lived experiences of communicators in fluid interactions. To address these theoretical lacunae, the present study employs proceduralized grounded theory (Corbin and Strauss, 2008) to inductively conceptualize the multifaceted nature of Chinese silence. By leveraging proceduralized grounded theory's constant comparative analysis, this research aims to construct a targeted, process-oriented framework that accounts for both situational adaptability and strategic flexibility of Chinese silence, and provide diagnostic tools for interpreting Chinese silence in increasingly complex multicultural encounters. To ensure analytical precision, this study distinguishes between competence-related silence arising from linguistic constraints and culturally-grounded silence conceptualized here as a dynamic praxis rooted in internalized norms and social agency. While the former is a universal phenomenon in L2 (second language) communication, this research focuses on the latter to explore how cultural frameworks and individual navigation transform silence from a mere functional barrier into a dynamic communicative practice.

## 2 Literature review

Silence is not merely the absence of speech but a communicative act that embodies multiple meanings and functions, and its interpretation varies widely across cultures (Nakane, 2007; Sun, 2016; Wang et al., 2020; Hayati and Sinha, 2024). While verbal communication tends to be explicit, silence is characterized by a wealth of unexpressed content that ordinarily far surpasses what is put into words (Poland and Pederson, 1998), whose interpretation rests on situational specifics and interlocutors' intended meanings (Ibrahim et al., 2023). Scholarly taxonomies differentiate silence into psycholinguistic, interactive, and sociocultural dimensions (Bruneau, 1973) or categorize it by turn-taking mechanics (Levinson, 1983) and intentionality (Kurzon, 1995). Beyond interpersonal discourse, the concept of Employee/Organizational Silence highlights how individuals strategically withhold information based on perceived risks or institutional climates (Morrison and Milliken, 2000; Dyne et al., 2003; Pinder and Harlos, 2001). Functionally, silence serves as a sophisticated pragmatic tool for politeness (Sifianou, 1997), display of power (Fivush, 2010), and relational management (Wu, 2019). It fulfills diverse linguistic roles, from referential function to metalanguage function (Ephratt, 2008), and can even alleviate tension, avoid conflict, and indirectly foster creativity (Baltezarević et al., 2022). As an active process of meaning-making embedded in relational and institutional contexts, the interpretation of silence is inherently contingent upon the broader cultural frameworks in which it is situated.

Existing scholarship has explained the cultural variations in silence through several macro-theoretical frameworks. Cross-cultural research frequently draws upon foundational theories to delineate the divergent cognitive and functional interpretations of silence, with Sino-Western comparisons providing critical theoretical insights. Studies consistently identify cultural dimensions as the primary determinants of silence's pragmatic utility. Integrating Hall's (1976) high and low context theory with Hofstede's (1980) cultural dimensions theory, scholars observe that low-context/individualistic cultures prioritize explicit expression, employ less silence, and often attribute negative connotations to it (Cheng and Tardy, 2009; Dodu-Savca, 2022; Gutiérrez and Paniagua, 2024). Conversely, in high-context/collectivist societies like China, where meaning is conveyed more implicitly, the use of silence is motivated to signal respect, avoid conflict, and maintain psychological equanimity (Gutiérrez and Paniagua, 2024; Turner, 2012). From the perspective of Politeness Theory (Brown and Levinson, 1987), silence serves as a face-saving politeness strategy, even regarded as the ultimate act of politeness by minimizing imposition (Sifianou, 1997) or equated with the “Don't do the FTA (Face-Threatening Act)” strategy when face threat is excessive (Nakane, 2007). These disparities can be anchored in divergent philosophical ontologies: Western rhetoric emphasizes agonistic self-assertion, whereas Confucian-Taoist thought prioritizes harmony, role order, and social equilibrium (Gu, 2004; Quan, 2015), providing a deeper cultural and theoretical rationale for the cross-cultural differences in silence.

Guided by these theoretical lenses, empirical research has extensively explored Chinese silence across diverse intercultural settings, including educational, workplace, and private domains, to uncover its specific manifestations, underlying motivations, and functional roles, as well as the resulting misinterpretations. In educational contexts, Hwang et al. (2002) found that Chinese students' reluctance to ask questions or provide feedback stems from face concerns and kiasuism (an obsessive fear of losing or falling behind others). Wen and Clément (2003) noted the cultural attributes of low willingness to communicate (WTC) among Chinese students, though Western educators often misperceive such reticence as inadequate language proficiency or low engagement (Zhu and Bresnahan, 2018). Jackson (2002) examined the psychological dimension of Chinese students' silence in English-medium business case discussions, identifying high levels of foreign language classroom anxiety as a key affective factor driving their reticence. Liu (2002) explored how Chinese students construct identity through silence via case studies, emphasizing silence's importance in intercultural communication. Recent research by Peng et al. (2023) confirmed a positive correlation between cultural distance and classroom silence, noting silence as protection reduces learning gains, while silence as power can enhance students' influence in classroom discussions, thereby increasing learning gains. Adding a phenomenological perspective, Yang et al. (2025) revealed that Chinese international students' classroom silence is a persistent study habit derived from their previous educational experiences; they tend to view lectures as collective “public time” and refrain from putting forward personal inquiries unless they consider the questions beneficial to the majority of the class.

In workplace and business settings, Western interlocutors often misinterpret Chinese silence as passivity, which in reality reflects cultural norms of deference and deliberation. Spencer-Oatey and Xing (2005) found that Chinese participants in Sino-British meetings accept silence due to high silence tolerance, power distance, language limitations, and relationship-building priorities, whereas British counterparts perceive it as awkward and uncomfortable. Verouden et al. (2018) reported that in Sino-Dutch scientific research cooperation, Dutch researchers often interpret the silence of Chinese collaborators as a lack of communication or insufficient investment, which leads to weakened trust between the two sides and hinders the progress of cooperation.

Beyond the domains of education and workplace, scholarly inquiry has also explored more nuanced social and private contexts, such as digital platforms and intimate relationships. Hu and Chen (2023) identified study-abroad experience, culture, relationships, and technology as key factors influencing Chinese international students' silence on social media. Cheng and Tardy (2009) found that Taiwanese (with higher individuals' interdependent self-construal) tend to use silence to protect their spouse's image, while Americans (with higher individuals' independent self-construal) often use it to control conflict and safeguard their own image. Pietikäinen (2018) further delineated five interactive functions of silence in intercultural couples' conflicts, confirming it as a strategy with interactive significance.

Despite these insights, existing literature on Chinese silence in intercultural communication still harbors notable limitations that this study seeks to address. A core shortcoming lies in fragmentation and the absence of a systematic analytical framework: while existing scholarship has extensively documented the manifestations, causes, and interpretations of silence in specific contexts, such as educational settings (e.g., Hwang et al., 2002) and business environments (e.g., Spencer-Oatey and Xing, 2005), these contributions remain largely fragmented within discrete empirical silos. Current analyses typically focus on surface-level behavioral patterns (e.g., reticence in inquiry) and their immediate antecedents (e.g., face-saving, power distance) within bounded scenarios, offering limited insight into the integrative mechanisms and core characteristics that underpin these behaviors. Additionally, the literature fails to elucidate the latent logical continuities that link a Chinese communicator's silence across disparate domains, such as the classroom or intimate discourse. There remains, therefore, a critical need to integrate these isolated findings into a holistic, systematic, and culturally-grounded analytical framework to better capture the cross-scenario consistency and psychological complexity of Chinese silence in intercultural communication. Furthermore, static perspectives predominate, lacking process-oriented perspectives: most existing research attributes silence to broad cultural dimensions (e.g., collectivism and high-context) nearly without exploring the dynamic psychological negotiation and process that occurs across different intercultural domains. Frameworks like Hofstede's are critiqued for static essentialism, ecological fallacy, and its limited capacity to capture contemporary shifts (Taras et al., 2011), stereotyping silence as an inevitable byproduct of collectivism or high-context cultures while overlooking its strategic agency and situational variability. To tackle this limitation, this study employs proceduralized grounded theory, a qualitative research methodology, to transcend description toward a participant-driven theoretical explanation of the dynamic and processual interactions grounded in empirical data. Through iterative coding and constant comparison of multi-source data, including semi-structured interviews, naturalistic observations, and textual artifacts, this research addresses two central questions: (1) What pragmatic characteristics does silence manifest for Chinese participants in intercultural communication? (2) How do these characteristics reflect the culturally embedded negotiation of face, power, and relational dynamics?

## 3 Methods

### 3.1 Research approach

This study employs a proceduralized grounded theory approach (Corbin and Strauss, 1990) to systematically analyze raw data through open, axial, and selective coding. Its core principles—contextual data collection, inductive conceptualization, and theoretical saturation (Li et al., 2019)—align with this study's goal of exploring the processual and dynamic characteristics of silence among Chinese participants in intercultural communication. Given the tacit, context-dependent nature of Chinese silence, proceduralized grounded theory enables an unconstrained investigation of its cultural internalization, contextual negotiation, and strategic enactment, supporting a data-driven understanding of silence as a dynamic communicative practice.

### 3.2 Data collection

Data were gathered through a triangulation of semi-structured interviews (*n* = 40: 30 Chinese, 10 foreign participants), naturalistic observations (45 sessions), and textual documents (*n* = 50). Chinese participants had substantial intercultural experience (e.g., studying or working abroad for at least 1 year or routinely interacting with foreign counterparts), regional diversity (participants were drawn from Northern, Southern, and Western China to reflect dialectal and cultural variation), and basic proficiency in English to ensure meaningful participation in intercultural exchanges; foreign participants had sustained engagement with Chinese counterparts (e.g., collaborating with Chinese colleagues for over 6 months). Naturalistic observations were conducted across academic, corporate, and diplomatic settings, prioritizing high-context and power-asymmetric interactions where silence was more likely to emerge. These thresholds may ensure that the observed silence patterns were less likely to be products of primary linguistic incompetence and more likely to reflect the active negotiation of cultural schemas and strategic communicative choices. In addition, textual materials were incorporated as secondary data to provide broader interpretive depth, including authoritative theoretical works on intercultural communication, philosophical and ethical texts on silence in traditional Chinese culture, and empirical studies on silence in Chinese intercultural contexts. Notably, these textual documents fulfill two distinct roles by informing the preliminary literature review while functioning as supplementary empirical evidence for methodological triangulation to corroborate and cross-validate primary interview and field data. Data collection and analysis proceeded iteratively: transcripts and field notes were coded, memos were written concurrently, with emerging concepts informing further sampling and questioning (theoretical sampling) (Birks and Mills, 2015). Of the 135 total data units collected, a primary coding corpus of 102 units was used for open and axial coding. A saturation corpus constituting approximately one quarter of each source type (33 units) was reserved for the theoretical saturation test.

### 3.3 Data analysis

Data analysis followed a systematic grounded theory approach, progressing from open and axial coding for concepts and categories labeling to selective coding to construct a coherent explanatory framework. This iterative process was supported by analytic memoing and managed via NVivo 14. To ensure interpretive rigor, two researchers performed independent coding with intercoder agreement quantified via Cohen's kappa. Disagreements were discussed and resolved to further improve reliability, and theoretical sampling continued until category saturation was reached, ensuring no new categories were identified in later coding.

## 4 Results

### 4.1 Open coding

Open coding is an interpretive process that analytically fractures raw data to identify and compare phenomena to generate conceptual labels and categories, enabling researchers to transcend preconceived biases and uncover new insights (Corbin and Strauss, 1990). The process was refined through the lens of theoretical sensitivity, which requires a synergy of analytic temperament and competence to maintain analytic distance and tolerate ambiguity, while developing theoretical insights and abstracting conceptual ideas from diverse data sources Holton, (2007). Throughout open coding, we maintained a reflexive yet neutral analytical stance to capture latent meanings and concepts behind raw data rather than relying on superficial interpretation, thereby enhancing in-depth understanding of intercultural interaction phenomena. Employing constant comparative analysis, we iteratively grouped raw data into higher-order conceptual clusters. Through this inductive process, 29 concepts emerged, which were further synthesized into 14 thematic categories serving as the foundation for the subsequent axial coding stage. Relevant coding results are shown in Table 1.

**Table 1:** Open coding in qualitative data analysis.

Categories	Concepts	Original statements
F1 Confucian “Cautious Speech” Norms	A1 verbal restraint is a marker of wisdom	I8 “My grandfather always said, ‘A wise man speaks little'—this mindset follows me even when working with foreigners.”
	A2 disaster comes from careless words	I11 “When chatting with foreign clients, I always remind myself to be cautious with words. One small slip can ruin a deal or damage relationships.”
F2 Collectivist Face Preservation	A3 ingroup face shielding	I7 “In multicultural teams, I feel it's better to remain silent when colleagues make a mistake. Directly addressing it might lose their face.”
	A4 outgroup face giving	I3 “During a business dinner, a foreign guest made a culturally inappropriate joke. I just kept quiet and smiled politely. I didn't want to make the situation more awkward.”
F3 High-Context Communication Schemas	A5 contextual meaning construction	T15 Chinese silence in intercultural contexts often carries implicit meaning, which can be a sign of respect, dissatisfaction, opposition, or cautious reservation through contextual cues.
	A6 non-verbal compensation	O4 When there's a sudden silence, the man tends to increase personal distance or shake his head in a way that almost fills the gap, like they're compensating for the lack of words.
F4 Power Distance Internalization	A7 internalized social hierarchy	I1 “When elders, superiors, and other senior foreigners are speaking, we usually never question their decisions. Even if we have differing opinions, we choose to remain silent because respecting elders is a rule we were taught from a young age.”
	A8 deference to authority figures	T23 Generally, Chinese overseas students seldom interrupt the teacher and always listen and wait patiently until the teacher has finished speaking. This is a basic form of respect for the teacher's authority.
F5 Moralization of Silence	A9 proverb activation	T34 “沉默是金” (silence is golden) is a popular Chinese idiom to indicate circumstances where it is better to say nothing than to speak.
	A10 virtue signaling	I18 “I feel that my strategic silence in intercultural communication shows self-control and maturity—a mark of true self-cultivation.”
F6 Power Distance Modulation	A11 modulation in vertical power contexts	I14 “When negotiating with German executives, I tend to take longer pauses to show respect and thoughtfulness, especially when I need to reflect on their point or give a more formal reply.”
	A12 modulation in horizontal power contexts	I15 “When talking with French colleagues of equal rank, I speak up quicker. I think interactions with them are more casual, and long silences can feel awkward.”
F7 Face-Threat Calculus	A13 appraisal of self-face threats	I2 “I often hesitate before answering a question because I'm worried that giving a wrong answer might expose a gap in my knowledge and make foreign teachers and classmates doubt my abilities, which would damage my image.”
	A14 appraisal of other-face threats	I6 “Before I contribute to a discussion and want to critique others' ideas, I often pause to think: will my comment help or hurt their face?”
F8 Cultural Schema Switching	A15 low-context adaptation	O8 Silence frequency decreases when interacting with American partners because they seem to prefer a more continuous flow of communication.
	A16 high-context reinforcement	O9 Participants deliberately prolong silence with Japanese counterparts because they seem to appreciate the space to think and value thoughtful pauses.
F9 Authority- Constructing Silence	A17 positioning pauses	O3 During the intercultural negotiation, the Chinese delegate intentionally paused for 5-7 seconds before presenting the core proposal, making the statement appear more deliberate and authoritative.
	A18 ritualistic withholding	O22 During the multicultural team meeting, the Chinese manager deliberately didn't respond to subordinates' suggestions.
	A19 multimodal markers	O23 During a critical negotiation pause, the Chinese manager steepled his hands while maintaining direct eye contact with the opposing party, conveying confidence and authority without uttering a word.
F10 Conflict- Averting Silence	A20 preemptive silence	I27 “When interacting with foreign friends, I would remain silent when sensitive topics like religious practices or political ideologies came up, fearing that expressing my stance might cause discomfort.”
	A21 de-escalation silence	I25 “My American friend kept raising his voice, but I didn't respond because I thought it would escalate the argument. Silence helped diffuse the tension and let things calm down.”
F11 Relational Investment Silence	A22 empathetic listening	O19 Told that her foreign friend had broken up with her boyfriend, the Chinese girl remained silent, leaning forward, gently nodding, and patting her on the shoulder to show understanding of her feelings and situation.
	A23 emotional bonding	I4 “In fact, sometimes, silence speaks louder than a thousand words because it carries the weight of companionship, understanding, and deep emotional resonance, which can draw the distance close.”
F12 Negative Reinforcement Loop	A24 cumulative silence efficacy	I9 “After a few successful interactions where silence helped me appear considerate, I became more comfortable using it.”
	A25 reinforced behavioral propensity	I16 “Keeping silent when I wasn't sure about the answer worked well because it saved me from embarrassment, so I'll do it again.”
F13 Code-Switching Fatigue	A26 cognitive- physiological overload	I21 “I feel mentally and physically drained, like my brain gets stuck during conversations with foreigners, and over time, I just become less willing to speak proactively.”
	A27 reduced verbal production	I28 “I noticed that when talking to my American classmates or friends for a long time, I tend to keep my tone flat, and I use fewer hand gestures to avoid overexpressing myself.”
F14 Habitual Fossilization	A28 habitual avoidance silence	O10 Once avoidance silence takes hold, it acts like an automatic response—some participants subconsciously stay quiet in intercultural interactions, even when there is no real threat.
	A29 habitual identity-aligned silence	I22 “I feel like silence has become part of my character—especially when interacting with foreigners. Staying quiet just feels authentically Chinese now.”

I—Semi-structured interviews; O—Naturalistic observations; T—Textual artifacts.

### 4.2 Axial coding

Axial coding relates main categories to subcategories through a coding paradigm comprising conditions, context, strategies, and consequences while systematically validating these relationships against empirical data to enhance conceptual density (Corbin and Strauss, 1990). This phase involves analytically constructing connections between categories to represent the empirically observed processes found in the raw data, while systematically validating these relationships against the original evidence to enhance conceptual density. Moving beyond superficial categorical classification, this phase adopts a dialectical analytical approach to extract core relational components: causal conditions (e.g., cultural norms necessitating facework), intervening conditions (e.g., adaptive modulation of silence duration amid power differentials), behavioral strategies (e.g., strategic silence enactment for conflict prevention), and consequential outcomes (e.g., the formation of ego-protective psychological habits). These dimensions integrate fragmented phenomena into a systematic and dynamic relational network. This rigorous analytical process aids in discerning overarching patterns, relationships, and thematic consistencies that underpin the theoretical construction. In this study, the 14 initial categories led to the abstraction of four main categories. The details are presented in Table 2.

**Table 2:** Main categories of axial coding.

Main categories	Categories	Connotations
Z1 Culture-Driven Predispositions	F1 Confucian “Cautious Speech” Norms F2 Collectivist Face Preservation F3 High-Context Communication Schemas F4 Power Distance Internalization F5 Moralization of Silence	Confucian “Cautious Speech” Norms provide ethical grounding, Collectivist Face Preservation balances relational harmony, High-Context Communication Schemas convey implicit meanings non-verbally, Power Distance Internalization hierarchizes silence duration, and Moralization of Silence elevates silence to a cultural virtue. These intertwined dimensions form a cultural matrix that shapes Chinese silence in intercultural communication as a dynamic interplay of tradition, ethics, and social hierarchy.
Z2 Context-Mediated Negotiations	F6 Power Distance Modulation F7 Face-Threat Calculus F8 Cultural Schema Switching	Power Distance Modulation hierarchically calibrates silence duration, Face-Threat Calculus deploys silence to mitigate relational risks, and Cultural Schema Switching adapts silence patterns to interlocutors' communication styles. These sub-processes synergize to balance cultural predispositions with situational demands, enabling Chinese communicators to maintain cultural coherence while dynamically adjusting silence strategies across intercultural contexts.
Z3 Strategic Enactment of Silence	F9 Authority-Constructing Silence F10 Conflict-Averting Silence F11 Relational Investment Silence	Authority-Constructing Silence leverages prolonged pauses to reinforce hierarchical control, Conflict-Averting Silence tactically de-escalates tensions, and Relational Investment Silence fosters trust via attentive non-verbal cues. These strategies form a situational toolkit, enabling Chinese communicators to weaponize silence as a pragmatic tool—calibrated to authority assertion, conflict mitigation, and relational alignment based on interactional goals and contextual risks.
Z4 Ego-Protective Inertia	F12 Negative Reinforcement Loop F13 Code-Switching Fatigue F14 Habitual Fossilization	Negative Reinforcement Loop initially entrenches silence through anxiety reduction, Code-Switching Fatigue perpetuates silence as a cognitive shortcut under depleted mental resources, and Habitual Fossilization rigidifies silence into identity alignment. These sub-mechanisms form a self-reinforcing cycle: avoidance-driven rewards reduce adaptive motivation, cognitive exhaustion locks in silence as the default behavior, and neural habituation transforms it into an identity-laden reflex, ultimately sustaining silence as a maladaptive yet persistent communicative inertia.

### 4.3 Selective coding

**Figure 1:**
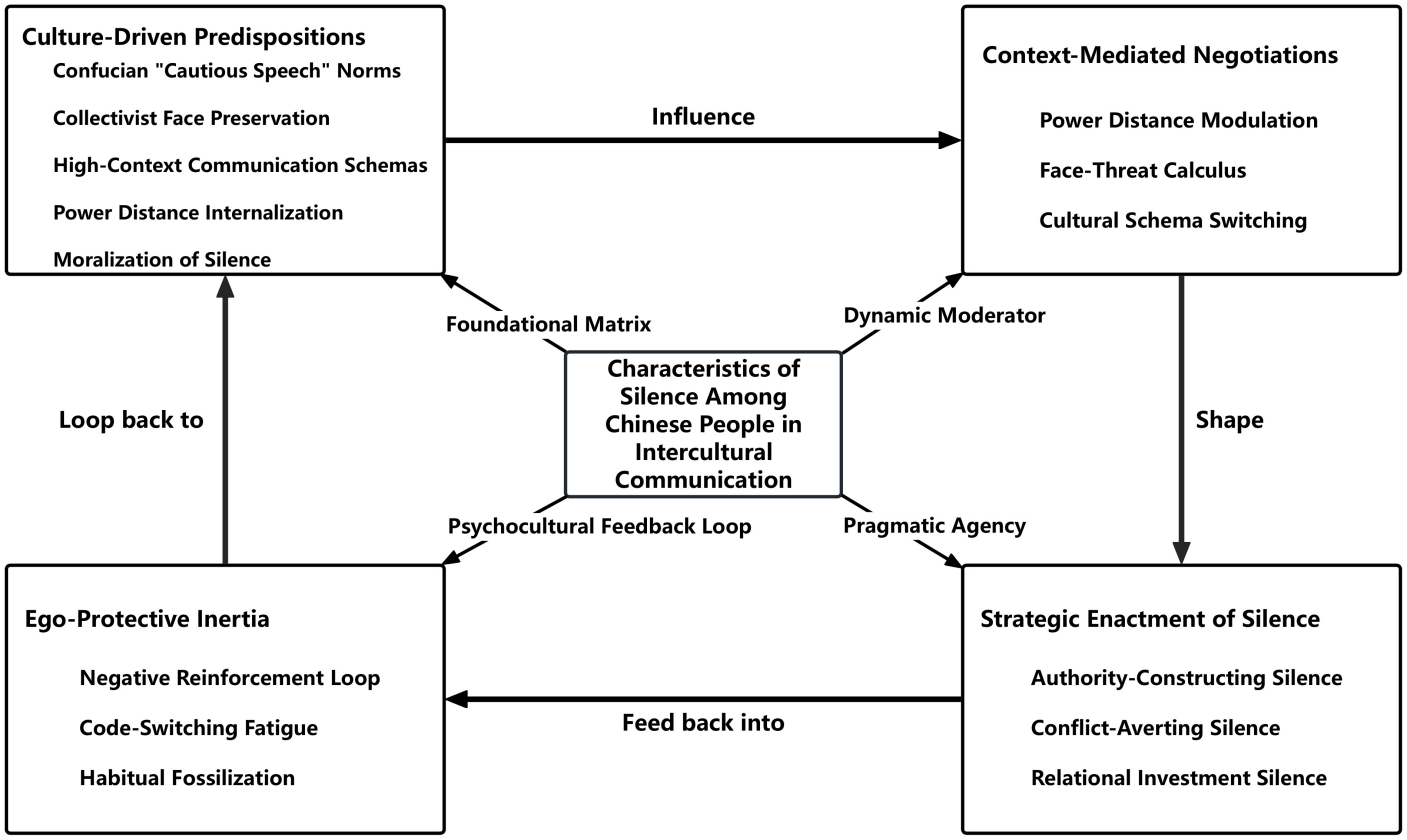
A model of characteristics of silence among Chinese people in intercultural communication.

Selective coding is the process by which all categories are unified around a core category representing the central phenomenon, facilitating the integration of theoretical relationships and the refinement of descriptive details (Corbin and Strauss, 1990). It is also termed “theoretical integration” within the analytical procedures of grounded theory (Corbin and Strauss, 2008). Following (Holton 2007), the core category is identified for its centrality, integrative capacity, and explanatory power. In the present study, the core category identified through selective coding is “Characteristics of Silence Among Chinese People in Intercultural Communication,” which serves as the conceptual nucleus around which other subcategories and concepts are systematically organized and integrated. To further elucidate this core category, a “storyline” is constructed: The phenomenon of Chinese communicators' silence in intercultural encounters unfolds as a dynamic ecosystem characterized by a dialectical interplay between cultural structure and individual agency. Culture-Driven Predispositions establish the foundational matrix, providing the normative gravity and semiotic repertoire that position silence as a culturally sanctioned default. Rather than acting as a deterministic constraint, this structural foundation is actively navigated and recalibrated through Context-Mediated Negotiations, which adjust silence behaviors in real-time based on situational demands. Within this negotiated space, silence transforms into Strategic Enactment, utilized as a purposeful pragmatic tool to achieve specific intercultural objectives. Crucially, the recurring success of these agentic choices, coupled with the cognitive demands of adaptation, feeds back into Ego-Protective Inertia, forming a feedback loop that reinforces the habitual use of silence, ultimately sustaining and shaping the core characteristics of silence as a persistent yet dynamically negotiated practice within intercultural communication. The proposed theoretical model is depicted in Figure 1.

### 4.4 Theoretical saturation test

To ensure the robustness of the emergent model, this study implemented a systematic theoretical saturation test. Following a split-data strategy, one-quarter of the raw data was reserved and subsequently reintroduced after the preliminary model construction to verify if any novel concepts or relationships would emerge. When the data collection is no longer useful for illustrating the phenomenon under study, the data saturation is reached (Cao et al., 2019). This iterative process was facilitated by NVivo 14, which monitored code frequency and density to identify the point of diminishing returns. To further enhance credibility, the study employed triangulation, integrating multiple data sources and researcher perspectives, alongside member validation, seeking feedback from participants to confirm the model's resonance. The final model was deemed theoretically saturated, demonstrating a comprehensive and stable framework of the characteristics of Chinese silence in intercultural communication.

## 5 Model interpretation

### 5.1 Culture-Driven Predispositions as the foundational matrix

The axial code “Culture-Driven Predispositions” elucidates how deeply internalized cultural frameworks systematically predispose Chinese communicators to employ silence as a culturally sanctioned default in intercultural encounters. Rather than an immutable trait, this predisposition functions as a multifaceted communicative framework that emerges from the interplay between historical cultural legacies and contemporary intercultural communication.

At its core, this predisposition operates through an interdependent network of cultural subsystems that shape the dynamic silence practices of Chinese communicators. Confucian “cautious speech” norms establish the ethical bedrock. Core tenets such as “verbal restraint is a marker of wisdom” (nè yán mǐn xíng 讷言敏行) and “disaster comes from careless words” (huò cóng kǒu chū 祸从口出) articulate a normative injunction toward verbal restraint. In this research, multiple interviewees acknowledged an ingrained tendency to exercise cautious speech in daily communication. One participant stated, “My grandfather always said, ‘A wise man speaks little'—this mindset follows me even when working with foreigners” (I8), reflecting the Confucian maxim that “the superior man is diligent in his work and careful in speech” (Gu, 2004, p.62). Speech is framed not as an autonomous right but as a moral act, to be exercised prudently and with ethical forethought. This normative framing conditions silence as a sign of maturity and rationality.

In collectivist moral orders that prize relational harmony (hé xié 和谐) and mutual face (miàn zi 面子), silence is socialized as a normatively appropriate default—a taken-for-granted way to align the self with group interests and to maintain the moral texture of interaction. For instance, consider the ingroup face shielding scenario illustrated by one participant's statement: “In multicultural teams, I feel it's better to remain silent when colleagues make a mistake. Directly addressing it might lose their face” (I7). By choosing not to directly point out errors, individuals avoid causing public embarrassment or humiliation, thereby preserving the collective image and internal cohesion of the group. Such group-oriented silence and face-maintaining preferences are not isolated situational choices, but rooted in long-standing traditional values shaped by historical inheritance. Centuries of feudal practice consequently preserved and transmitted collectivist values, embedding them so deeply that they continue to shape contemporary Chinese mindsets and behaviors (Wang, 2019). Within this ethos, overt contradiction, public correction, or unsolicited self-display risks disturbing the group's moral equilibrium; by contrast, measured taciturnity signals deference, restraint, and respect. Thus, silence functions primarily as a group-protective convention: it preserves shared dignity and cohesion before any individual cost-benefit reasoning is engaged.

Crucially, these norms are operationalized through high-context communication schemas, wherein silence is culturally loaded with meanings decipherable through shared contextual knowledge—a system that often clashes with low-context interlocutors' expectations of explicit verbalization. In participants' actual interaction, silent pauses are rarely meaningless blank spaces; instead, they convey implicit attitudes such as polite deference, mild disagreement, and thoughtful deliberation (T15). Chinese participants also tended to compensate for linguistic vacancy through non-verbal adjustments, including interpersonal distance regulation and head movements during silent moments (O4). These observable communicative behaviors echo Hall's (1976) high-/low-context dichotomy. Chinese high-context culture relies heavily on culturally shared background knowledge and situational cues, so that contextual framing outweighs the informational load carried by explicit linguistic code (Quan, 2015). Within this communicative ecology, silence functions as a nuanced paralinguistic resource that carries context-dependent meanings, such as showing obedience to elders, respecting others' wisdom, implying disagreement without direct confrontation, or pausing to collect thoughts (Gu, 2004). For interlocutors from low-context cultures, who value explicit and unambiguous verbal communication, this silence might be misinterpreted as indifference, apathy, or even hostility (Dodu-Savca, 2022), leading to feelings of uncertainty and discomfort, as they grow anxious about the direction of the conversation and contemplate changing the topic to avoid losing the other party's interest (Li, 2011).

Chinese people's silence is also shaped by deeply entrenched hierarchical norms. Participants clearly reported restraining their own opinions and remaining silent when interacting with hierarchical superiors and senior members. One respondent noted, “When elders, superiors, and other senior foreigners are speaking, we usually never question their decisions. Even if we have differing opinions, we choose to remain silent because respecting elders is a rule we were taught from a young age” (I1). This internalized social hierarchy manifests distinctly in intercultural settings: Chinese overseas students seldom interrupt foreign teachers, waiting patiently until the lecture ends to respond, a practice rooted in deference to authority (T23). Hofstede's (1980) power-distance dimension helps situate this expectation at the culture level: China's endorsement of status asymmetry means that superiors' prerogatives are taken for granted and publicly foregrounded (Sun, 2016). Such silence, as a default respect token, preserves hierarchy in intercultural classrooms or negotiations, becoming a recognizable behavioral pattern even for foreign interlocutors.

Meanwhile, silence in Chinese culture is moralized and elevated to the status of virtue. The maxim “silence is golden” (chén mò shì jı̄n 沉默是金) crystallizes the culturally endorsed belief that, under certain circumstances, refraining from speech is preferable to speaking (T34). The prevalence of proverbs related to silence underscores how silence, as a moral and virtuous act, is deeply embedded in the Chinese collective consciousness. It functions simultaneously as a moral imperative and a visible index of personal virtue, signaling maturity, self-discipline, and ongoing self-cultivation. While nearly 75% of participants invoked “silence is golden” to legitimize their reticence, the remaining 25%, predominantly younger individuals with long-term overseas residency, expressed a significant detachment from this traditional maxim. For instance, one interviewee who moved to the UK for undergraduate studies noted that proverbs related to silence had negligible influence on her communicative choices, as her deep assimilation into Western academic culture favored assertive verbalization over contemplative restraint. This divergence underscores that the moralization of silence is undergoing a detraditionalization process among those at the forefront of intercultural flux.

By anchoring silence in this multilayered cultural matrix, this predisposition serves as the ontological foundation for the other three core codes. It sets the cultural baseline for contextual silence adjustments, endows strategic silence with ethical and social legitimacy, and provides the cultural origin for the formation of ego-protective inertial silence. This foundational predisposition shapes the activation logic, behavioral boundaries, and functional orientation of the other codes, underpinning the entire dynamic ecosystem of Chinese intercultural silence and framing it as a culturally intentional praxis where tradition, ethics, and social structure converge to guide communicative choices.

### 5.2 Context-Mediated Negotiations as a dynamic moderator

The axial code Context-Mediated Negotiations captures the adaptive recalibration of silence behaviors in response to situational demands, revealing the intrapsychic negotiation where Chinese communicators exert agency to balance their deeply ingrained cultural predispositions against the immediate, often conflicting, demands of intercultural exigencies, including the communicative expectations of non-Chinese interlocutors, situational norms, and relational dynamics. This code underscores silence not as a static cultural trait but as a contextually contingent practice, shaped by power dynamics, face threats, and cultural familiarity. Its role as a defining feature lies in its capacity to explain the fluidity of silence patterns across scenarios, challenging monolithic views of Chinese communicative behavior.

Power Distance Modulation represents the situational activation and adjustment of silence, functioning as an active negotiation between internalized Confucian hierarchical norms and real-time intercultural power dynamics. Rather than mere passive behavioral tuning, individuals mobilize silence as a semiotic resource to encode and perform role-based expectations while weighing the status of non-Chinese interlocutors. This process involves calibrating response latency, turn-entry timing, and channel selection (e.g., shifting critique to private side chat) to align cultural ethics with situational demands. In vertical power contexts, asymmetric encounters, prolonged pauses, and deferred topic shifts render abstract hierarchies visible (I14); conversely, in horizontal power contexts, shortened gaps reorient silence toward signaling equal participation (I15). Ultimately, Power Distance Modulation embodies the fusion of cultural semiotics and situational agency, demonstrating that silence is a dynamic tool for navigating the fluidity of intercultural status asymmetry.

Face-Threat Calculus also represents a situated interpersonal negotiation: Chinese speakers proactively evaluate, in real time, the likely face costs and benefits of speaking vs. withholding, negotiating between their own face needs, the face needs of their non-Chinese interlocutors, and the specific relational variables at play—intimacy, audience size/publicity, task stakes, prior history, and perceived linguistic/cultural competence. Silence here is not a default convention but a protective buffer when anticipated relational damage exceeds an acceptable threshold. Within Ting-Toomey's (1988) face-negotiation framework, this appraisal integrates concern for both positive face and negative face for self and other. For instance, I2 highlights self-oriented face-threat considerations, revealing that silence functions as a buffer to avoid the personal embarrassment that might arise from a misstep, thereby protecting the individual's self-esteem and public image. Similarly, in assessing the face-threat impact on others, I6 suggests that a reflective pause before criticism or differing opinions may minimize the risk of publicly embarrassing others, thus preserving group harmony and mutual respect. In short, Face-threat Calculus may help Chinese people evaluate and manage interpersonal risk in intercultural encounters.

Cultural Schema Switching captures the active negotiation between Chinese interlocutors' internalized high-context silence norms and the communicative logic of their non-Chinese counterparts. Grounded-theory analysis shows that participants consciously modulated both the duration and communicative function of pauses when engaging non-Chinese speakers, effectively toggling between cultural frames of reference as an act of intentional adaptation. Aligning with Nakane's (2007) contention that the analysis of intercultural interaction should shift from merely identifying cultural differences to investigating how interlocutors contextually negotiate their cultural norms through modification, assertion, or accommodation, participants were found to dynamically calibrate their use of silence. In low-context settings where clarity and continuous verbal flow are paramount, they often curtailed silences to reduce perceptions of disengagement (O8); conversely, in high-context encounters they often prolonged pauses, deploying silence as a relational resource that signals respect, reflective consideration, and social alignment (O9). Thus, silence functions not as a rigid cultural residue but as a dynamic, context-sensitive tool for smoothing interaction and preempting miscommunication.

These sub-processes interact through a dynamic negotiation, mediated by the active agency of Chinese communicators: deep-seated cultural scripts exert gravitational inertia, while immediate contextual factors generate adaptive pressures, and individuals proactively negotiate between these two forces to shape their silence practices. This code bridges the foundational cultural predispositions and the strategic use of silence, as its dynamic adjustment of silence lays the behavioral and semantic groundwork for intentional strategic enactment. In essence, Context-Mediated Negotiations define the boundaries of adaptability for silence, while Strategic Enactment transforms this adaptive behavior into purposeful agency, forging a dialectical relationship between situational fit and pragmatic goals.

### 5.3 Strategic Enactment of Silence as pragmatic agency

Building on the contextually adaptive silence shaped by Context-Mediated Negotiations, the axial code Strategic Enactment of Silence further elevates silence from situationally adjusted behavior to purposeful communicative agency, illuminating how Chinese interlocutors leverage the semiotic resources and adaptive outcomes of contextual negotiations to strategically employ silence for specific intercultural objectives.

This purposeful communicative agency is materialized via three strategic silence types: Authority-Constructing Silence refers to the practice where leaders and senior figures deploy deliberate positioning pauses—as derived from naturalistic observation data (O3), typically lasting 5 to 7 s—to assert dominance and command respect. This practice echoes ancient imperial rhetorical patterns, reminiscent of the yù yán (御言) tradition, where measured silence was used as a marker of superior status. For example, the deliberate pause during an intercultural negotiation served to make the ensuing statement appear more considered, deliberate, and ultimately authoritative. Another facet is ritualistic withholding that occurs when communicators deliberately choose not to respond or offer immediate feedback, thereby signaling deference or a thoughtful stance in a group setting (O22). The third type involves the use of multimodal markers—non-verbal signals that accompany periods of silence to further enhance their communicative impact. This combination of physical posture and sustained gaze conveyed a strong sense of confidence and authority without the need for words (O23).

Conflict-Averting silence operates as a strategic mechanism to mitigate intercultural tensions through two primary manifestations. One key manifestation is preemptive silence, where Chinese individuals intentionally refrain from commenting on sensitive topics to avoid triggering discomfort or disagreement. For instance, one participant explained: “When interacting with foreign friends, I would remain silent when sensitive topics like religious practices or political ideologies came up, fearing that expressing my stance might cause discomfort” (I27). This proactive decision to withhold immediate response demonstrates how silence is employed as a shield against potential conflict. By choosing not to voice opinions on divisive subjects, the Chinese communicator preemptively averts the risk of escalating tensions. Another critical dimension is de-escalation silence, which functions as a reactive cooling-off mechanism during episodes of heightened emotional intensity. By deliberately withdrawing from inflammatory exchanges, individuals utilize silence to defuse immediate friction rather than exacerbate it (I25). This strategic reticence aligns with negative politeness principles, prioritizing the minimization of imposition and the preservation of negative face. Ultimately, such conflict-averting practices demonstrate a heightened sensitivity to interpersonal harmony, transforming silence into a constructive tool for navigating cultural differences and fostering respectful intercultural environments.

Relational Investment Silence emphasizes attentive presence and emotional attunement, especially within high-context, relationship-oriented cultures. It entails deliberately refraining from verbal intrusion to create psychological space for the other party, conveying empathy, respect, and solidarity through non-verbal means. In emotionally charged moments, silence serves as a medium of care, allowing space for feelings, demonstrating deep listening, and fostering trust. As observed in a representative interaction (O19), upon learning of a foreign peer's relationship dissolution, a Chinese participant maintained prolonged silence while leaning forward, nodding gently, and patting the friend's shoulder. This non-discursive response effectively communicated deep understanding and emotional alignment, illustrating how silence can be strategically deployed as a powerful tool for relational maintenance.

These three strategic types—authority construction, conflict aversion, and relational investment—establish silence as a sophisticated, context-calibrated toolkit for pragmatic agency. It demonstrates how Chinese communicators actively leverage reticence to assert control, manage interpersonal risk, and fulfill socio-relational obligations, thereby challenging assumptions of passivity and highlighting the intentional, functional role of silence in achieving specific, positive outcomes in intercultural settings.

### 5.4 Ego-protective Inertia as a psychocultural feedback loop

The formation of Ego-Protective Inertia originates from the long-term iteration of intentional communicative strategies, which is fully supported by participants' firsthand narratives and qualitative findings. Ego-Protective Inertia elucidates the self-perpetuating mechanisms that solidify silence into fossilized response patterns. In this context, the “Ego” is conceptualized not as a detached, individualistic construct, but as a culturally embedded entity—a “relational ego” that seeks to maintain psychological equilibrium and social worth within a hierarchy. This mechanism addresses a puzzle that Chinese communicators maintain silence despite recognizing its interpersonal costs. It reflects a strategic yet defensive self-positioning where the ego prioritizes the avoidance of perceived threat to its cultural integrity. This phenomenon is driven by three interlocking reinforcement pathways from situational anxiety relief to cognitive exhaustion and finally to automatic habit formation.

Negative Reinforcement Loop, where silence is conditioned through the immediate reduction of communicative anxiety. In high-stakes or face-threatening interactions, refraining from speech provides psychological relief by avoiding potential verbal missteps or conflict. This relief activates neural reward systems, reinforcing silence as an emotionally safe and socially commendable choice within Chinese cultural frameworks that prioritize face. For instance, as one participant (I16) reflected, the tactical decision to remain silent when uncertain served as an effective shield against potential embarrassment, with this positive outcome directly incentivizing the recurrence of such behavior in future encounters. Consequently, this cycle diminishes the motivation to invest the cognitive resources necessary for intercultural adaptation, laying the groundwork for subsequent exhaustion.

Code-Switching Fatigue drives a retreat to silence as a cognitive shortcut when mental resources deplete. The dual challenge of processing unfamiliar linguistic cues and adhering to different cultural norms demands significant cognitive effort, overwhelming the brain's capacity to maintain active engagement. Drawing on Sweller's (1988) Cognitive Load Theory (CLT), human working memory capacity for active cognitive processing is finite. When the demands of linguistic decoding and cultural adjustment saturate this capacity, individuals shift toward silence as a low-effort survival strategy. This cognitive drain diverts energy away from active verbal production and paralinguistic expressiveness (I28), reducing the interaction to its most basic, silent form. Repeated reliance on this shortcut, fueled by negative reinforcement, increases the frequency of silent responses, gradually stripping them of conscious strategic intent.

Habitual Fossilization reflects the transformation of silence from a contextually strategic choice into an internalized, automatic response. Several participants acknowledged that silence has gradually developed into an ingrained personal trait, especially in interactions with foreign interlocutors, and maintaining a reserved, taciturn manner has come to be regarded as an inherent reflection of their Chinese cultural identity (I22). In intercultural encounters, one's cultural group identity becomes particularly salient. As outlined in Self-Categorization Theory (SCT), when a specific social identity becomes salient, individuals undergo psychological depersonalization, shifting from idiosyncratic personal identity to behaving in terms of shared group norms (Turner and Reynolds, 2012). This stage functions as the model's solidification mechanism: it utilizes the individual's perceiver readiness, the relatively enduring cultural knowledge about the self, as a psychological resource that, through repeated reinforcement, renders silence a culturally appropriate and right response. By bridging short-term situational adaptations with long-term cultural identity, this process converts temporary silence into a stable, cross-situational behavioral norm. For instance, in encounters with elders, the salience of the Chinese junior identity triggers an instinctive return to reticence. Silence here is no longer a mere polite gesture but an authentic enactment of filial propriety and social identity, explaining why it persists even when maladaptive, as it has become a variable judgment based on categorization-in-context that is now tied to core self-perception.

This self-perpetuating cycle elucidates the paradox in intercultural perception: while Western interlocutors may interpret Chinese silence as a passive lack of engagement or competence, for the Chinese communicator, it represents a sophisticated, albeit counter-intuitive, strategy of psychological preservation. By theorizing this inertia, the study reveals that Chinese silence is not a mere communicative void, but a dynamic, processual negotiation of the “relational ego” striving for equilibrium in misaligned environments. Understanding this mechanism is vital for fostering intercultural empathy, as it shifts the focus from judging silence as a linguistic failure to recognizing it as an adaptive response, thereby paving the way for deeper cultural adaptation and more effective mutual accommodation.

## 6 Conclusion

### 6.1 Research findings

This proceduralized grounded theory investigation of 135 intercultural episodes reveals Chinese silence not as a monolithic cultural trait but as a dynamic, contextually mediated practice shaped by four interdependent dimensions: Culture-Driven Predispositions, Context-Mediated Negotiations, Strategic Enactment, and Ego-Protective Inertia. The first axial category underscores how Confucian collectivism, hierarchical deference, and mianzi consciousness structurally condition silence. Rather, Context-Mediated Negotiations reveal how situational variables—power differentials, perceived cultural threat, and interlocutor familiarity—modulate silence's duration and semiotic weight. Crucially, silence emerges as Strategic Enactment rather than passive withdrawal: participants selectively deployed reticence to assert hierarchical authority, avert intercultural conflict, and nurture empathic relational bonds. Paradoxically, this strategic agency coexists with Ego-Protective Inertia, where silence functions as a psychological buffer in cross-cultural misalignment. The interplay between these dimensions reveals a sophisticated communicative ecosystem in which silence can be proactively employed as a strategic device for relational management, yet may also gradually evolve into an identity-linked habit through a self-perpetuating feedback loop. These findings underscore that Chinese silence is a dynamic and process-oriented communicative practice, reflecting the ongoing negotiation between individual agency and a culturally embedded sense of psychological security.

### 6.2 Research contributions

This study offers a diagnostic framework for interpreting Chinese silence in intercultural settings, helping educators, managers, and diplomats distinguish strategic silence from disengagement. By identifying when silence functions as deference, emotional regulation, or rapport-building, the model reduces misattributions in communication. It also informs intercultural competence training by encouraging Western interlocutors to cultivate an appreciation for purposeful pauses, while simultaneously empowering Chinese speakers to bridge potential gaps through verbal cues or metacommunicative signals. The framework can be applied in international education, business, and diplomacy to enhance cultural sensitivity and communicative effectiveness. Moreover, it supports inclusive pedagogy by validating silence as a legitimate form of participation, shifting classroom expectations away from speech-centric norms. Overall, the model may promote mutual understanding and adaptive strategies in multicultural societies.

### 6.3 Research limitations and future directions

While the model offers a nuanced account of Chinese intercultural silence, several limitations should be acknowledged. The sample primarily comprises urban-educated professionals engaged in Western-dominant communication contexts, which may overrepresent strategic silence and underrepresent silence motivated by relational harmony or emotional inhibition in non-professional or rural settings. Furthermore, the dataset draws heavily on China-West interactions, potentially limiting the model's transferability to South-South intercultural encounters—such as Sino-African, Sino-Southeast Asian, or Sino-Middle Eastern interactions—where postcolonial power dynamics may shape silence differently. Thirdly, as this study is rooted in qualitative grounded theory, it prioritizes processual depth over statistical generalization. The causal links between the main categories were derived through qualitative narrative logic and the coding paradigm. Hence, the relative influence and functional weight of each formative mechanism requires further quantitative empirical testing. To test the framework's boundary conditions, future research should adopt intersectional and stratified sampling strategies, taking into account variables such as geographic origin (urban/rural), intercultural experience (novice/advanced), generational cohort (e.g., Gen Z/Gen X), and digital literacy. Crucially, mixed-methods and longitudinal designs are needed to track the evolution of silence practices over time. Such studies may quantitatively verify the model's stability and reveal how cultural inertia interacts with rapid globalization and digital shifts, providing a more robust empirical basis for the transition from intentional strategy to habitual behavior.

## Data Availability

The original contributions presented in the study are included in the article/Supplementary material, further inquiries can be directed to the corresponding author.
