# Correction: Characteristics of silence among Chinese people in intercultural communication: a proceduralized grounded theory analysis

**DOI:** 10.3389/fpsyg.2026.1895094

**Published:** 2026-06-22

**Authors:** Hongya Fan, Weiqian Xiang

**Affiliations:** School of Foreign Languages, Shanxi University, Taiyuan, Shanxi, China

**Keywords:** Chinese people, intercultural communication, multicultural environments, proceduralized grounded theory, silence

There were 2 mistakes in Table 2 as published. First, the category F1 Confucian “Cautious Speech” norms had a capitalization formatting error; the correct term is F1 Confucian “Cautious Speech” Norms.

**Table 2:** Main categories of axial coding.

Main categories	Categories	Connotations
Z1 Culture-Driven Predispositions	F1 Confucian “Cautious Speech” Norms F2 Collectivist Face Preservation F3 High-Context Communication Schemas F4 Power Distance Internalization F5 Moralization of Silence	Confucian “Cautious Speech” Norms provide ethical grounding, Collectivist Face Preservation balances relational harmony, High-Context Communication Schemas convey implicit meanings non-verbally, Power Distance Internalization hierarchizes silence duration, and Moralization of Silence elevates silence to a cultural virtue. These intertwined dimensions form a cultural matrix that shapes Chinese silence in intercultural communication as a dynamic interplay of tradition, ethics, and social hierarchy.
Z2 Context-Mediated Negotiations	F6 Power Distance Modulation F7 Face-Threat Calculus F8 Cultural Schema Switching	Power Distance Modulation hierarchically calibrates silence duration, Face-Threat Calculus deploys silence to mitigate relational risks, and Cultural Schema Switching adapts silence patterns to interlocutors' communication styles. These sub-processes synergize to balance cultural predispositions with situational demands, enabling Chinese communicators to maintain cultural coherence while dynamically adjusting silence strategies across intercultural contexts.
Z3 Strategic Enactment of Silence	F9 Authority-Constructing Silence F10 Conflict-Averting Silence F11 Relational Investment Silence	Authority-Constructing Silence leverages prolonged pauses to reinforce hierarchical control, Conflict-Averting Silence tactically de-escalates tensions, and Relational Investment Silence fosters trust via attentive non-verbal cues. These strategies form a situational toolkit, enabling Chinese communicators to weaponize silence as a pragmatic tool—calibrated to authority assertion, conflict mitigation, and relational alignment based on interactional goals and contextual risks.
Z4 Ego-Protective Inertia	F12 Negative Reinforcement Loop F13 Code-Switching Fatigue F14 Habitual Fossilization	Negative Reinforcement Loop initially entrenches silence through anxiety reduction, Code-Switching Fatigue perpetuates silence as a cognitive shortcut under depleted mental resources, and Habitual Fossilization rigidifies silence into identity alignment. These sub-mechanisms form a self-reinforcing cycle: avoidance-driven rewards reduce adaptive motivation, cognitive exhaustion locks in silence as the default behavior, and neural habituation transforms it into an identity-laden reflex, ultimately sustaining silence as a maladaptive yet persistent communicative inertia.

Second, the main category Z2 Context-Mediated Negotiarions was misspelled; the correct term is Z2 Context-Mediated Negotiations.

The corrected Table 2 appears below.

The reference for (Birks and Mills, 2015) was erroneously written as:

Birks, M., and Mills, J. (2015). *Grounded Theory: A Practical Guide, Edn., 2nd ed*. Londond: SAGE.

It should be:

Birks, M., and Mills, J. (2015). *Grounded Theory: A Practical Guide, 2nd Edn*. London: Sage.

The original version of this article has been updated.

