# Supplementary material for: Characteristics of silence among Chinese people in intercultural communication: a proceduralized grounded theory analysis
Source: Front Psychol. 2026 May 21;17:1840234. doi: 10.3389/fpsyg.2026.1840234 (PMC13235302; doi:10.3389/fpsyg.2026.1840234)
Supplement: Supplementary file 1 [file Supplementary_file_1.docx]

***Supplementary Material***

# **Semi-structured Interview Questions**

1. What values in Chinese culture do you think have influenced your silent behavior in intercultural communication?

2. In what situations did you choose to be silent when communicating with foreigners? Can you describe the situation?

3. How do you understand the role of silence in intercultural communication? Is it positive or negative?

4. In intercultural communication, what is the usual reason for your silence? Is it cultural differences or psychological problems?

5. Is your own desire for silence when interacting with foreigners generally voluntary or forced?

6. Do you think Chinese people tend to be more silent or outspoken when communicating with people from different cultures? Why?

7. What do you think is the role of silence in avoiding conflict and promoting interpersonal relationships in intercultural communication? Can you give an example?

8. When communicating with foreigners/Chinese people, have you ever intentionally chosen silence to avoid conflict or avoid misunderstanding? Can you give an example?

9. When you have encountered misunderstandings in intercultural communication, have you ever chosen to defuse the situation through silence?

10. Do you consider silence an avoidance strategy? If so, is it effective in intercultural communication?

11. Do you choose silence to express specific emotions in intercultural communication? For example, anger, sadness, anxiety, etc.

12. Have you ever chosen to be silent in intercultural communication for fear of damaging your image?

13. Have you ever observed the reaction of foreigners to your silent behavior? How do they perceive Chinese people’s silence?

14. Have you ever been misunderstood or confused by foreigners because of your silence? If so, can you share your experience at that time?

15. Do you think silence helps to show your respect for others in intercultural communication?

16. How do you think Chinese people use silence in intercultural communication to adapt to different cultural ways of communication?

17. Do you choose to remain silent in intercultural communication because you don’t want to hurt the other person’s face?

18. Might silence convey your disapproval of a topic or dissatisfaction with someone?

19. When you choose to be silent in intercultural communication, what is your body language and expression usually like?

20. Are the patterns and durations of silence different when communicating with people from different countries?

21. How do you think maintaining proper silence in front of foreigners has affected you?

22. If silence brings positive feedback, you will choose silence again next time in the same situation, right?

23. Do you have any comments or suggestions about the questions and discussions in this interview? Are there any other phenomena of silence that you think are important to add?
